# Oncologic impact of delaying radical prostatectomy in men with intermediate- and high-risk prostate cancer: a systematic review

**DOI:** 10.1007/s00345-021-03703-8

**Published:** 2021-05-28

**Authors:** Ekaterina Laukhtina, Reza Sari Motlagh, Keiichiro Mori, Fahad Quhal, Victor M. Schuettfort, Hadi Mostafaei, Satoshi Katayama, Nico C. Grossmann, Guillaume Ploussard, Pierre I. Karakiewicz, Alberto Briganti, Mohammad Abufaraj, Dmitry Enikeev, Benjamin Pradere, Shahrokh F. Shariat

**Affiliations:** 1 Department of Urology, Comprehensive Cancer Center, Vienna General Hospital, Medical University of Vienna, Währinger Gürtel 18-20, 1090 Vienna, Austria; 2 Institute for Urology and Reproductive Health, Sechenov University, Moscow, Russia; 3 Men’s Health and Reproductive Health Research Center, Shahid Beheshti University of Medical Sciences, Tehran, Iran; 4 Department of Urology, The Jikei University School of Medicine, Tokyo, Japan; 5 Department of Urology, King Fahad Specialist Hospital, Dammam, Saudi Arabia; 6 Department of Urology, University Medical Center Hamburg-Eppendorf, Hamburg, Germany; 7 Research Center for Evidence Based Medicine, Tabriz University of Medical Sciences, Tabriz, Iran; 8 Department of Urology, Dentistry and Pharmaceutical Sciences, Okayama University Graduate School of Medicine, Okayama, Japan; 9 Department of Urology, University Hospital Zurich, Zurich, Switzerland; 10 Department of Urology, La Croix du Sud Hospital, Quint Fonsegrives, Toulouse, France; 11 Cancer Prognostics and Health Outcomes Unit, Division of Urology, University of Montreal Health Center, Montreal, Canada; 12 Department of Urology, Vita Salute San Raffaele University, Milan, Italy; 13 Division of Urology, Department of Special Surgery, Jordan University Hospital, The University of Jordan, Amman, Jordan; 14 Department of Urology, Weill Cornell Medical College, New York, NY USA; 15 Department of Urology, University of Texas Southwestern, Dallas, TX USA; 16 Department of Urology, Second Faculty of Medicine, Charles University, Prague, Czech Republic; 17 Karl Landsteiner Institute of Urology and Andrology, Vienna, Austria; 18 European Association of Urology Research Foundation, Arnhem, Netherlands

**Keywords:** Prostate cancer, Deferred, Radical prostatectomy, PCa, RP, COVID-19

## Abstract

**Purpose:**

To summarize the available evidence on the survival and pathologic outcomes after deferred radical prostatectomy (RP) in men with intermediate- and high-risk prostate cancer (PCa).

**Methods:**

The PubMed database and Web of Science were searched in November 2020 according to the PRISMA statement. Studies were deemed eligible if they reported the survival and pathologic outcomes of patients treated with deferred RP for intermediate- and high-risk PCa compared to the control group including those patients treated with RP without delay.

**Results:**

Overall, nineteen studies met our eligibility criteria. We found a significant heterogeneity across the studies in terms of definitions for delay and outcomes, as well as in patients’ baseline clinicopathologic features. According to the currently available literature, deferred RP does not seem to affect oncological survival outcomes, such as prostate cancer-specific mortality and metastasis-free survival, in patients with intermediate- or high-risk PCa. However, the impact of deferred RP on biochemical recurrence rates remains controversial. There is no clear association of deferring RP with any of the features of aggressive disease such as pathologic upgrading, upstaging, positive surgical margins, extracapsular extension, seminal vesicle invasion, and lymph node invasion. Deferred RP was not associated with the need for secondary treatments.

**Conclusions:**

Owing to the different definitions of a delayed RP, it is hard to make a consensus regarding the safe delay time. However, the current data suggest that deferring RP in patients with intermediate- and high-risk PCa for at least around 3 months is generally safe, as it does not lead to adverse pathologic outcomes, biochemical recurrence, the need for secondary therapy, or worse oncological survival outcomes.

## Introduction

The rapid spread of coronavirus disease 2019 (COVID-19), caused by the novel severe acute respiratory syndrome coronavirus-2 (SARS-CoV-2), has had a profound impact on the worldwide health care systems [1]. The clinical practice of healthcare workers and systems worldwide has substantially changed secondary to the present pandemic. Individual monitoring through precision diagnostics, interdisciplinary team boards, and therapy adjustments is an essential part of the adjustment of medical workers to the COVID-19 pandemic, urology being no exception [2, 3].

Delays in diagnosis and treatment of cancer patients can have an adverse impact on disease outcomes. In some cases, the benefit of ensuring a timely delivery of a definitive anti-cancer treatment outweighs the potential risk of a COVID-19 infection. Hence, several medical societies, including urologic societies, have introduced clinical guidelines to advise physicians about appropriate treatment decisions during the current pandemic [4]. General recommendations for surgical procedures should consider resource allocation as well as operation room (OR) and intensive care unit (ICU) capacities as well as the risk of COVID-19 transmission while minimizing its impact on disease outcomes. Therefore, evidence-based recommendations are needed to appropriately categorize a disease state as either high or low priority. In general, radical treatment can be deferred justifiable only if delays are unlikely to affect cancer outcomes [5]. The impact of treatment delay on disease outcome should be measurable to make the appropriate decision regarding the urgency and priority of a specific surgical/medical intervention.

However, it was reported that the number of radical prostatectomies (RP) in March–July 2020 was reduced by approximately 50% compared to the baseline period of March–July 2019 [6]. Although treatment delay in low-risk PCa is expected to have no or minimal impact on the disease outcomes, especially with the implementation of active surveillance, prolonged delays in treatment of intermediate or high risk PCa without regular monitoring could potentially result in disease progression [7]. Nevertheless, high level evidence on the subsequent risk of delaying RP during COVID-19 pandemic in patients with intermediate- and high-risk PCa is still lacking. We hypothesized that there is no evidence in the existing literature that a treatment delay of up to 3 months has a significant oncological impact in men affected by PCa. To test our hypothesis, we conducted a systematic review of the literature on the impact of deferring RP in intermediate- and high-risk PCa patients during the period before the COVID-19 pandemic.

This systematic review aimed to summarize the available evidence on the survival and pathologic outcomes after deferred RP in men with intermediate- and high-risk PCa.

## Methods

### Literature search

This systematic review was conducted according to the Preferred Reporting Items for Systematic Reviews and Meta-analyses (PRISMA) statement [8]. The study protocol was registered a priori on the International Prospective Register of Systematic Reviews (PROSPERO; Registration ID CRD42020224178).

The PubMed and Web of Science databases were searched in November 2020 to identify studies reporting on the survival and pathologic outcomes after deferred RP in men with intermediate- and high-risk PCa. A comprehensive systematic literature search was independently performed by two authors. The keywords used in our search strategy included: "delayed" OR "deferred" AND "radical prostatectomy" AND "prostate cancer" AND "intermediate risk" AND "high risk". In addition, we searched the references of selected studies for potentially relevant articles. The main outcomes of interest were survival and pathologic outcomes.

After removing duplicates, two independent reviewers screened the titles and abstracts. Any citation which either reviewer thought should be included or unclear for inclusion was identified for full text screening. Subsequently, full texts of eligible articles were reviewed for final inclusion and data extraction. In cases of disagreement, the authors consulted with the co-authors, and final decisions were reached by consensus.

### Inclusion and exclusion criteria

We included studies that reported on survival and pathologic outcomes after deferred RP in men with intermediate- and high-risk PCa. The PICO (population, intervention, control, and outcomes) in this study was the following: patients treated with deferred RP for intermediate- and high-risk PCa compared to control group including those patients treated with RP without delay. The outcomes were PCa recurrence, survival and pathologic outcomes.

We excluded reviews, letters, editorials, animal studies, study protocols, case reports, meeting abstracts, replies from authors, brief correspondence, and articles not published in English. Furthermore, we excluded studies that did not provide data regarding the oncologic outcomes. References of all papers included were scanned for additional studies of interest.

### Data extraction

Data extracted from each study were independently extracted by two reviewers. Extracted data included the following: first author’s name, publication year, study design, demographics characteristics including age range, sample size, risk group, PSA level, median follow up, median time from diagnosis to RP, definition of RP delay. Subsequently, the hazard ratios (HR) or odds ratio (OR) and 95% confidence intervals (CI) associated with each outcome were retrieved.

## Results

### Search results

**Fig. 1:**
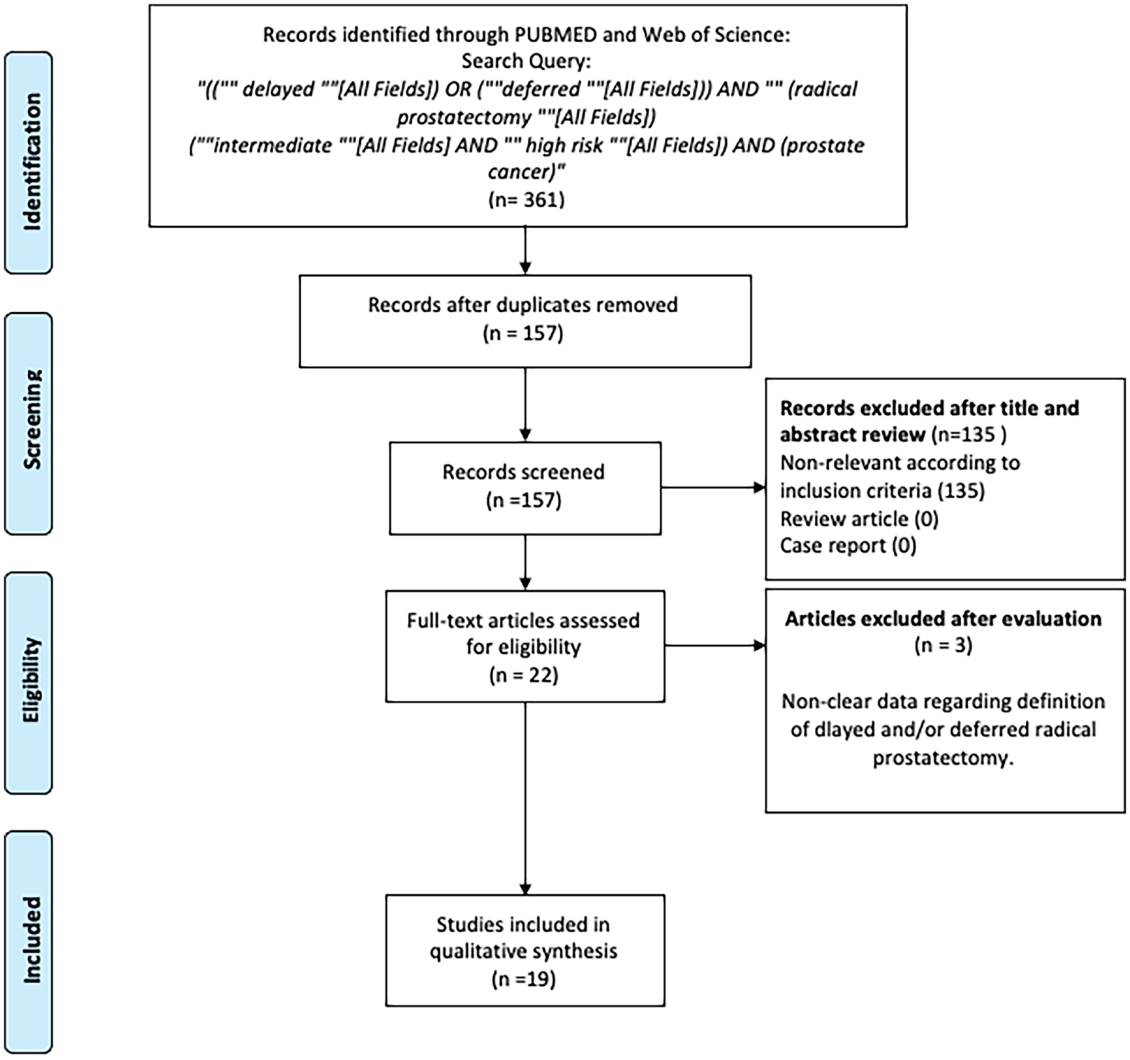
Selection process of the articles to assess the survival and pathologic outcomes after deferred radical prostatectomy in men with intermediate- and high-risk PCa

The literature search identified 361 unique references. Among them, 204 records were removed due to duplication, and 135 articles were excluded due to unrelated outcomes during the screening process (Fig. 1). Of the 22 full-text articles assessed for eligibility, three were excluded based on the selection criteria.

Nineteen studies were finally included in the present systematic review [9–27]. Table 1 summarizes the studies and their main findings. A few studies included low-risk patients along with intermediate and high-risk patients without performing separate analyses in terms of risk groups [11, 13, 17, 18, 24, 27]. We made the decision to include these studies in the qualitative analysis.

**Table 1:** Characteristics of included studies

Author, publication year	Study design	Number of patients	Age, years, median, range or IQR)	Risk group, stage	PSA, ng/ml, median (range or IQR)	Follow-up, median (range or IQR)	Median time from diagnosis to RP (range)	Definition of RP delay	Main results
Aas 2018 [25]	R	5163	62 (39–77)	Low (28.2%); intermediate (42.9%); high (28.9%)	NR	7.9 years (0–15)	93 days (1–180)	Stratified by interval: ≤ 60 days (16.5%); 61–90 days (30.7%); 91–120 days (25.5%); 121–180 days (27.3%)	No association was found between RP-interval and PCSM in the intermediate-or high-risk groups Increasing RP-interval did not increase the rate of adverse histological outcomes (upgrading, upstaging, PSM) or incidence of RP-failure
Abern 2012 [9]	R	1561	NR	Low (52%); intermediate (48%)	NR	53 months (IQR 25–86)	NR	Stratified by interval: ≤ 3 months (60%); 3–6 months (30%); 6–9 months (6%); > 9 months (4%)	For intermediate-risk, delays > 9 months were significantly related to BCR (HR: 2.10, *p* = 0.01), PSM (OR: 4.08, *p* < 0.01), and ECE (OR: 6.68, *p* = 0.045)
Anil 2018 [23]	R	248	64.2	Low (49.2%); intermediate (25.4%); high (25.4%)	9.96	16.1 months (2–72)	NR	Stratified by interval: ≤ 60 days (43.1%); 61–120 days (45.6%); ≥ 120 days (11.3%)	No significant difference between the groups in terms of BCR (*p* = 0.06) or additional treatment (*p* = 0.1) ECE increased significantly in the intermediate-risk group with longer surgical delay (*p* = 0.044)
Balakrishnan 2019 [19]	P	1916	61 (IQR 56–65)	Low and intermediate risk:GG1 (86.6%), GG2 with 1 high grade core (6.9%), GG2 with 2 or more high grade cores(6.5%)	5.50 (IQR 4.31–7.38)	66 (IQR 42–93)	27 months (IQR 15.5–46.5)	After AS	GG2 with 2 or more high grade cores at diagnosis was associated with an increased risk of recurrence compared to GG1 disease (HR 3.29, 95% CI 1.49–7.26, *p* < 0.01). GG2 disease with 1 high grade core did not significantly differ from GG1
Berg 2015 [10]	R	2,212	Mean 60.77 (SD 7.2)	Low (34.9%); intermediate (54.6%); high (10.4%)	NR	39 months (IQR 12–82)	64 days (48–90)	Stratified by interval: ≤ 90 days (75.7%); 91–180 days (24.3%); > 180 days (2.7%)	Significant increases in the proportion of adverse pathological outcomes were found beyond 60 days for patients with Gleason 7 and PSA > 20 (*p* = 0.032), and 30 days for patients with Gleason 8–10 and PSA 11–20 (*p* = 0.041)
Cooperberg 2011 [17]	P	466	NR	Low and intermediate CAPRA risk	NR	37.5 (IQR 27–60)	19.5 (IQR 14–36)	After AS	No statistically significant differences regarding adverse histological outcomes (upstaging, PSM, ECE) (all *p* > 0.05)
Diamand 2020 [26]	R	926	66 (IQR 61–70)	Intermediate (67%); high (33%)	8.2 (5–12)	26 months (IQR 10–40)	3 months (2–5)	3 months—cutoff	Delay was not significantly associated with upgrading (OR 0.98, 95% CI 0.94–1.02, *p* = 0.3), nor to LNI (OR 0.88, 95% CI 0.77–1.01, *p* = 0.07), pathological locally advanced disease (OR 1, 95% CI 0.97–1.03, *p* = 0.8), or need for adjuvant therapy (OR 0.96, 95% CI 0.84–1.11, *p* = 0.6), or BCR (HR 0.97, 95% CI 0.91–1.04, *p* = 0.6)
Filippou 2015 [11]	R	3,372	Mean 60.6 (SD 6.9)	Low and intermediate	4.9 (IQR 4.0–6.1)	40 months (7–166)	20 months (6–148)	RP > 6 months after diagnostic biopsy	Immediate RP had lower probability of adverse pathology than delayed RP (OR 0.34, 95% CI 0.21–0.55)
Fossati 2016 [14]	R	2,653	66 (60–70)	Low (35%); intermediate (50%); high (15%)	6.5 (4.8–9.8)	56 months (IQR 26–92)	2.8 months (IQR 1.6–4.7)	NR	Time from biopsy to RP was significantly associated with an increased risk of BCR (HR 1.02, *p* = 0.0005) and CR (HR 1.03, *p* = 0.0002)
Ginsburg 2020 [20]	R	128,062	63 (IQR 58–67)	Intermediate and high risk	6.3 (IQR 4.8–9.7)	NR	3 months (IQR 2–4)	Stratified by interval: 0–3 months (73.2%), 4–6 months (23.7%), 7–9 months (2.5%), 10–12 months (0.6%)	Delay up to 12 months was not significantly associated with odds of adverse pathology, upgrading, node positive disease or post-RP secondary treatments
Godtman, 2018 [18]	R	132	64 (IQR 61–67)	Low (85%); intermediate (14%); high (1%)	4.1 (IQR 3.4–5.5)	10.9 years (IQR 7.5–14.5)	1.9 years (IQR 1.2–4.2)	After AS	39% experienced at least 1 unfavorable pathology feature at RP. The 10-year prostate specific antigen relapse-free survival was 79.5%
Gupta 2019 [22]	R	2303	62 (IQR 57–66)	Intermediate, high: GG3 (54%), GG4 (26%), GG5 (20%)	6.0 (IQR 4.5–9.1)	3 years (IQR 2–5)	NR	< 3 months (72%), 3–6 (28%)	There was no significant difference in rates of adjuvant therapy, PSM, EPE, SVI, or LNI There was no significant difference in 2- and 5-year BCRFS There was no significant difference in 2-, 5-, and 10-year MFS
Korets 2011 [24]	R	1568	60 (IQR 56–66)	Low, intermediate, high risk	NR	NR	45 days (36–847)	Stratified by interval: ≤ 60 days (70%); 61–90 days (19.3%); > 90 days (10.7%)	A delay of > 60 days was not associated with adverse pathological findings at surgery or worse BCR
Morini, 2017 [21]	R	908	61.5	Low (47.4%); intermediate (40.8%); high (11.9%)	Mean 7.88 (0.02–53.55)	Mean 4 years	Mean 191.0 (30.0–941.0)	Stratified by interval: ≤ 6 months (56.5%); 6–12 months (38.5%); > 12 months (5.1%)	No time interval correlated with poor oncological outcomes (including BCR) in all risk groups
Nesbitt 2020 [27]	R	332	Mean 63.1 (SD 6.4)	Low, intermediate, high risk	Mean 7.2 (SD 5.6)	NR	Mean 125.7 days (SD 70.0)	> 90 days	Time between biopsy and surgery was not associated with adverse outcome except for pathological ECE or pT3 disease (*p* = 0.04)
Patel 2019 [12]	R	2728	Mean 60.2 (SD 6.9)	GG1 (39%), GG2 (45%), GG3 (10%), GG4 (3%), GG5 (2.4%)	6.0 (IQR 4.7–8.1)	NR	83 days (61–109)	< 2 months and then at monthly intervals of up to 6 months	Delays of up to 6 months were not associated with an increased risk of upgrading, ECE, SVI, PSM, or LNI
Zakaria, 2020 [16]	R	1057	Mean 60.9 (SD 6.5)	Low (36.0%); intermediate (53.6%); high (10.3%) UCSF-CAPRA risk	Mean 6.8 (SD 3.6)	NR	NR	NR	Cohort analysis showed correlation between CAPRA-score difference and wait time (Pearson correlation: *r* = − 0.062; *p* = 0.044)
Zanaty 2017 [15]	R	619	NR	Low (35%); intermediate (50%); high (15%)	NR	22 months	153 days	NR	Surgical wait time was positively correlated to BCR for high-risk group (*p* = 0.001). On threshold analysis, cutoff was found to be 90 days
Westerman 2019 [13]	R	7350	Mean 61.5 ± 7.1	NCCN Risk Group: low (53.6%); intermediate (37.7%); high (8.7%)	Mean 7.1 ± 7.3	7.1 years (IQR 4.2–11.7)	61 days (IQR 37–84)	≤ 3 weeks, 4–6 weeks, 7–12 weeks, 12–26 weeks, and > 26 weeks	High risk men waiting more than 6 months had higher rates of BCR (HR: 3.38, *p* = 0.05)

*BCR* biochemical recurrence; *BCRFS* biochemical recurrence-free survival; *CI* confidential interval; *CR* clinical recurrence; *ECE* extracapsular extension; *HR* hazard ratio; *GG* Gleason grade; *IQR* interquartile range; *LNI* lymph node invasion; *MFS* metastasis-free survival; *NCCN* National Comprehensive Cancer Network; *OR* odds ratio; *P* prospective; *PCa* prostate cancer, *PCSM* prostate cancer-specific mortality; *PSM* positive surgical margins; *R* retrospective; *RP* radical prostatectomy; *SD* standard deviation; *SVI* seminal vesicle invasion

## Definition of delay

We found a significant heterogeneity across the studies in terms of delayed RP definitions. Most of the studies used 3-month intervals between diagnosis and RP [9, 10, 20–22]; three studies used 2-month intervals [23–25]. Diamand et al. and Nesbitt et al. used a cutoff of 3 months [26, 27], while Filippou et al. used a delay of > 6 months after diagnostic biopsy [11]; Patel et al. used an interval < 2 months and then monthly intervals up to 6 months [12]. Westerman et al. used the following intervals: ≤ 3, 4–6, 7–12, 12–26, and > 26 weeks [13]. Fossati et al., Zakaria et al., and Zanaty et al. used time between diagnosis and RP as a continuous variable [14–16]. Three studies reported that the delay groups were followed with an AS protocol [17–19]. Korets et al. excluded men on AS [24].

## Definition of outcomes

Two studies reported data on survival outcomes, particularly on prostate cancer-specific mortality (PCSM) [25] and metastasis-free survival (MFS) [22]. Nine studies reported data on biochemical recurrence (BCR) [9, 13, 15, 21–24, 26]. Fourteen studies reported data on adverse pathologic outcomes such as upgrading, upstaging, positive surgical margins (PSM), extracapsular extension (ECE), seminal vesicle invasion (SVI), lymph node invasion (LNI), etc.[9–12, 16, 20–27]. Five studies reported data on the need of secondary therapy after surgery [20, 22, 23, 26, 27].

## Observations of outcome

### Biochemical recurrence

Five studies found no significant impact of treatment delay on BCR [21–24, 26], while four studies found that treatment delay had an unfavorable impact in intermediate- [9] and high-risk [13–15] PCa patients (Table 2).

**Table 2:** Biochemical recurrence along the studies

Author, publication year	Results according to definition of delay				
Anil 2018 [23]	≤ 60 days (2 months) 27.6%	61–120 days (2–4 months) 30.6%	≥ 120 days (4 months) 0%	*p* = 0.06	
Korets 2011 [24]	≤ 60 days (2 months) HR 1	61–90 days (2–3 months) HR 1.26; 95% CI 0.94–1.70; *p* = 0.12	> 90 days (3 months) HR 1.13; 95% CI 0.73–1.31; *p* = 0.43		
Abern 2012 [9]		≤ 3 months HR 1	3–6 months HR 1.00; 95% CI 0.76–1.33; *p* = 0.989	6–9 months HR 0.72; 95% CI 0.40–1.28; *p* = 0.262	> 9 months HR 2.19; 95% CI 1.24–3.87; *p* = 0.01
Morini 2017 [21]			≤ 6 months HR 1	6–12 months HR 0.691; 95% CI 0.466–1.026; *p* = 0.067	> 12 months HR 0.416; 95% CI 0.139–1.243; *p* = 0.116
Westerman 2019 [13]	≤ 3 weeks IR: HR 1 High risk: HR 1	4–6 weeks IR: HR 1.07; 95% CI.88–1.31; *p* = 0.51 High risk: HR 1.15; 95% CI 0.86–1.54; *p* = 0.34	7–12 weeks IR: HR 1.11; 95% CI 0.91–1.36; *p* = 0.31 High risk: HR 1.35; 95% CI 0.98–1.87; *p* = 0.07	12–26 weeks IR: HR 0.98; 95% CI 0.75–1.29; *p* = 0.9 High risk: HR 1.16; 95% CI 0.71–1.91; *p* = 0.55	> 26 weeks IR: HR 0.99; 95% CI 0.58–1.68; *p* = 0.97 High risk: HR 3.03; 95% CI 1.05–8.78; *p* = 0.04
Diamand 2020 [26]	3-month delay was not significantly associated with BCR (HR 0.97, 95% CI 0.91–1.04, *p* = 0.6)				
Fossati 2016 [14]	Time from biopsy to RP was significantly associated with an increased risk of BCR (HR 1.02, *p* = 0.0005) A significant increased risk of BCR after approximately 12 months was observed in high-risk group				
Zanaty 2017 [15]	Surgical wait time was positively correlated to BCR for high-risk group (*p* = 0.001) On threshold analysis, cutoff was found to be 90 days				
Gupta 2019 [22]	There was no significant difference in 2- and 5-year BCRFS between both intermediate- and high-risk patients who had RP < 3 months vs. 3–6 months after diagnosis in terms of GG: GG3: 78% vs. 83% and 69% vs. 66%, respectively, *p* = 0.6; GG4: 68% vs. 74% and 51% vs. 57%, respectively, *p* = 0.4; GG5: 58% vs. 74% and 48% vs. 54%, respectively, *p* = 0.2				

*BCR* biochemical recurrence; *BCRFS* biochemical recurrence-free survival; *CI* confidential interval; *GG* Gleason score; *HR* hazard ratio; *IR* intermediate risk; *RP* radical prostatectomy

Abern et al. suggested 9 months as a threshold for association with BCR (HR 2.10, *p* = 0.01) among men with intermediate-risk PCa [9]. For high-risk patients, Westerman et al. found that higher rates of BCR were observed with more than 6-month delay in RP (HR 3.38, *p* = 0.05) [13], while Fossati et al. reported significantly increased risk of BCR after approximately 12 months [14]. At the same time, Zanaty et al. reported a cutoff surgical waiting time of 90 days (3 months) for an increase in the rate of BCR (*p* = 0.001) [15].

In contrast, Diamand et al. observed no association between RP delay of more than 3 months after diagnosis with BCR (HR 0.97, 95% CI 0.91–1.04, *p* = 0.6) in both intermediate- and high-risk patients [26]. In agreement with these results, Gupta et al. reported that there was no significant difference in 2- and 5-year biochemical recurrence-free survival (BCRFS) in both intermediate- and high-risk patients who had RP < 3 months vs. those who had between 3 and 6 months after diagnosis [22].

In summary, owing to inconsistencies in findings among studies, the impact of deferred RP, in patients with intermediate-or high-risk PCa, on BCR is still controversial. However, most of the studies reported that around 3-month delay was not significantly associated with BCR, especially in intermediate-risk PCa patients (Table 2).

### Survival outcomes

Two studies found no significant impact of treatment delay on survival outcomes (PCSM and MFS) [22, 25]. Aas et al. reported no association between RP-interval and PCSM in the intermediate-or high-risk groups in a study of 5163 patients with time from diagnosis to treatment stratified by intervals: ≤ 60, 61–90, 91–120 and 121–180 days [25]. Among 2303 intermediate- and high-risk patients, Gupta et al. reported that there was no significant difference in 2-, 5-, and 10-year MFS between patients who had RP < 3 months vs. 3–6 months after diagnosis and according to Gleason grade group (GG3: 98, 92, and 84% vs. 97, 95, and 91%, respectively, *p* = 0.4; GG4: 97, 90, and 72% vs. 94, 91, and 81%, respectively, *p* = 0.8; GG5: 89 and 81% vs. 91 and 71%, respectively, *p* = 0.9) [22]. Taken together, at least 3-month deferred RP does not seem to affect the oncologic survival outcomes in patients with intermediate- and/or high-risk PCa, but only two studies with different definition of delay assessed these outcomes.

### Pathologic outcomes

Seven studies found no significant impact of treatment delay on pathologic findings [12, 20–22, 24–26], while five studies found that treatment delay had an unfavorable impact in intermediate- [9–11, 16, 23] and high-risk [10] PCa patients (Table 3). One study reported inconsistent correlations between time from diagnosis to RP and different pathologic outcomes [27].

**Table 3:** Pathologic outcomes along the studies

Author, publication year	Results according to definition of delay					
Anil 2018 [23]		≤ 60 days (2 months)	61–120 days (2–4 months)	≥ 120 days (4 months)		
	EPE	Ref	OR 2.250; 95% CI 1.029–4.918; *p* = 0.042	OR 0.694; 95% CI 0.206–2.341; *p* = 0.556		
	SVI	Ref	OR 0.396; 95% CI 0.143–1.092; *p* = 0.073	OR 0.162; 95% CI 0.024–1.111; *p* = 0.064		
	PSM	Ref	OR 1.569; 95% CI 0.735–3.351; *p* = 0.244	OR 1.674; 95% CI 0.509–5.513; *p* = 0.397		
	LVI	Ref	OR 1.500; 95% CI 0.362–6.213; *p* = 0.576	OR 1.640; 95% CI 0.110–24.540; *p* = 0.720		
Korets 2011 [24]		≤ 60 days (2 months)	61–90 days (2–3 months)	> 90 days (3 months)		
	ECE	Ref	OR 1.03; 95% CI 0.78–1.35; *p* = 0.84	OR 0.95; 95% CI 0.69–1.04; *p* = 0.07		
	SVI	Ref	OR 0.91; 95% CI 0.69–1.20; *p* = 0.49	OR 0.91; 95% CI 0.62–1.11; *p* = 0.45		
	PSM	Ref	OR 1.13; 95% CI 0.85–1.51; *p* = 0.38	OR 1.06; 95% CI 0.66–1.41; *p* = 0.86		
	LVI	Ref	OR 0.87; 95% CI 0.56–1.33; *p* = 0.53	OR 0.99; 95% CI 0.59–1.68; *p* = 0.68		
	UG	Ref	OR 1.11; 95% CI 0.83–1.48; *p* = 0.48	OR 1.08; 95% CI 0.78–1.44; *p* = 0.96		
Aas, 2018 [25]	PSM	≤ 60 days (2 months) IR: 29.6 HR: 27.7	61–90 days (2–3 months) IR: 30.4 HR: 34.4	91–120 days (3–4 months) IR: 23.1 HR: 33.9	121–180 days (4–6 months) IR: 21.1 HR: 35.7	*p* = 0.02 *p* = 0.46
Ginsburg 2020 [20]			≤ 3 months	4–6 months	7–9 months	10–12 months
	AP		Ref	OR 0.98; 95% CI 0.94–1.02; *p* = 0.31	OR 1.02; 95% CI 0.91–1.13; *p* = 0.773	OR 1.00; 95% CI 0.80–1.26; *p* = 0.98
	LVI		Ref	OR 1.02; 95% CI 0.93–1.12; *p* = 0.608	OR 0.91; 95% CI 0.68–1.22; *p* = 0.533	OR 1.06; 95% CI 0.65–1.74; *p* = 0.814
	UG		Ref	OR 1.00; 95% CI 0.95–1.05; *p* = 0.922	OR 1.09; 95% CI 0.95–1.24; *p* = 0.228	OR 1.06; 95% CI 0.82–1.37; *p* = 0.649
Abern 2012 [9]			≤ 3 months	3–6 months	6–9 months	> 9 months
	ECE		Ref	NR	NR	OR 6.68, 95% CI 1.04–42.77, *p* = 0.045
	PSM		Ref	OR 1.01; 95% CI 0.71–1.44; *p* = 0.941	OR 1.03; 95% CI 0.53–1.99; *p* = 0.929	OR 4.08; 95% CI 1.52–10.91; *p* = 0.005
Morini 2017 [21]				≤ 6 months	6–12 months	> 12 months
	ECE			9.9%	12.1%	10.6%
	SVI			6%	3.8%	2.1%
	PSM			34.1%	33.5%	31.2%
	LVI			2.7%	4.3%	2.1%
	UG			35.2%	40.2%	35.4%
Berg 2015 [10]	Significant increases in the proportion of adverse pathological outcomes were found beyond 60 days for patients with Gleason 7 and PSA > 20 (*p* = 0.032), and 30 days for patients with Gleason 8–10 and PSA 11–20 (*p* = 0.041)					
Diamand	3-month delay was not significantly associated with upgrading (OR 0.98, 95% CI 0.94–1.02, *p* = 0.3), LNI (OR 0.88, 95% CI 0.77–1.01, *p* = 0.07), pathological locally advanced disease (OR 1, 95% CI 0.97–1.03, *p* = 0.8)					
Filippou 2015 [11]	Immediate RP had a lower probability of adverse pathology than delayed RP > 6 months after diagnostic biopsy (OR 0.34, 95% CI 0.21–0.55) The rate of adverse pathology did not differ between immediate and delayed RP > 6 months in patients matched for pretreatment characteristics (OR 0.79, 95% CI 0.27–2.28)					
Gupta 2019 [22]	There was no significant difference in rates of PSM, EPE, SVI, or LNI in men who had RP < 3 months vs. 3–6 months after diagnosis in terms of GG: GG3: PSM: 22% vs. 21%, *p* = 0.7; EPE: 50% vs. 48%, *p* = 0.6; SVI: 13% vs. 11%, *p* = 0.3; LNI: 6% vs. 4%, *p* = 0.3; GG4: PSM: 19% vs. 21%, *p* = 0.7; EPE: 53% vs. 46%, *p* = 0.2; SVI: 13% vs. 13%, *p* = 1.0; LNI: 7% vs. 5%, *p* = 0.4; GG5: PSM: 34% vs. 32%, *p* = 0.7; EPE: 72% vs. 74%, *p* = 0.8; SVI: 32% vs. 33%, *p* = 0.8; LNI: 19% vs. 16%, *p* = 0.5					
Nesbitt 2020 [27]	Time between biopsy and surgery more than 90 days (3 months) was not associated with adverse outcomes (upgrading, PSM) except for pathological ECE or pT3 disease (*p* = 0.04)					
Patel 2019 [12]	Delays of up to 6 months were not associated with an increased risk of upgrading, ECE, SVI, PSM, or LNI					
Zakaria 2020 [16]	Cohort analysis showed correlation between CAPRA-score difference and wait time (Pearson correlation: *r* = − 0.062; *p* = 0.044)					

*Ap* adverse pathology; *CI* confidential interval; *ECE* extracapsular extension; *EPE* exctraprostatic extension; *GG* Gleason score; *HR* high risk; *IR* intermediate risk; *LNI* lymph node invasion; *OR* odds ratio; *PSM* positive surgical margins; *RP* radical prostatectomy; *SVI* seminal vesicle invasion; *UG* upgrade

Diamand et al. found that 3 months as a threshold of RP delay was neither associated with upgrading (OR 0.98, 95% CI 0.94–1.02, *p* = 0.3), LNI (OR 0.88, 95% CI 0.77–1.01, *p* = 0.07), nor upstaging (OR 1, 95% CI 0.97–1.03, *p* = 0.8) [26]. In agreement with these results, Gupta et al. indicated that there was no significant difference in rates of PSM, ECE, SVI, or LNI in men with intermediate- and high-risk PCa who had RP < 3 months vs. 3–6 months after diagnosis [22]. Moreover, Ginsburg et al. reported that RP delay even up to 12 months was not significantly associated with adverse pathologic outcomes in patients with intermediate- and high-risk disease [20].

Some studies obtained controversial results. Abern et al. noted that if RP was delayed > 9 months, PSM (OR 4.08, *p* < 0.01) and ECE (OR 6.68, *p* = 0.045) rates were higher among men with intermediate-risk PCa [9]. More strict cutoff was obtained by Berg et al. in a study with median time from diagnosis to treatment of 64 days (48–90); the risk of adverse pathologic findings increased beyond 60 days for patients with intermediate-risk disease (*p* = 0.032) and 30 days for patients with high-risk disease (*p* = 0.041) [10]. Interestingly, Nesbitt et al. reported that a time between biopsy and surgery of more than 90 days (3 months) was not significantly associated with pathologic upgrading or higher rate of PSM. However, they found a higher proportion of patients with ≥ pT3 disease in the group who had RP > 90 days after the biopsy (*p* = 0.04) [27].

Summing up, according to the currently available literature, deferring RP in patients with intermediate- or high-risk PCa does not seem to lead to a worsening in pathologic outcomes. Ten of twelve studies agreed that around 3-month delay is unlikely to affect pathologic outcomes (Table 3).

### Adjuvant therapy

Five studies found no significant impact of treatment delay on need for adjuvant therapy [20, 22, 23, 26, 27]. Diamand et al. found that RP delay of more than 3 months after diagnosis was not significantly associated with the need for adjuvant therapy (OR 0.96, 95% CI 0.84–1.11, *p* = 0.6) in intermediate- and high-risk patients [26]. Ginsburg et al. reported that RP delay up to 12 months was not significantly associated with post-RP secondary treatments in patients with both intermediate- and high-risk disease [20]. Gupta et al. reported that there was no significant difference in rates of adjuvant therapy in men with intermediate- and high-risk PCa who had RP < 3 months vs. 3–6 months after diagnosis [22]. Nesbitt et al. reported that waiting more than 90 days from biopsy to surgery did not put men at an increased risk of the need for postoperative treatment such as radiation therapy [27]. Anil et al. did not report a significant difference between the intervals between prostate biopsy and RP (≤ 60, 61–120, ≥ 120 days) in terms of additional treatment (*p* = 0.1) [23]. Thereby, deferred RP does not seem to be associated with the need for secondary treatment in patients with intermediate- or high-risk PCa. Although definition of a delayed surgery was different among all above studies, a delay around 3 months was shown to be safe in all of them in terms of the need for adjuvant therapy.

## Clinical implications and discussion

During the current COVID-19 pandemic, it is critical for clinicians to consider the impact of delayed RP for individual PCa patients [28]. COVID-19 should not result in missing the ideal time frame for treatment, especially in men with high-risk disease. For this reason, we conducted a systematic review summarizing the available evidence on the impact of deferring RP in men with intermediate- and high-risk PCa.

According to the currently available literature, there is no clear, reproducible, and significant difference in oncologic and survival outcomes, including PCSM and MFS, between patients who underwent immediate RP and those who underwent deferred RP after approximately 3 months. Although it seems that the delaying of RP for 3–6 months after diagnosis in patients with intermediate- and high-risk PCa was not associated with worsening of the survival outcomes, the current data are not sufficient enough to reliably consider this as an evidence. This is mainly due to the limited numbers of studies assessing the impact of this delay on the disease outcome. Furthermore, BCR and adverse pathologic findings were assessed in almost all the studies included in this systematic review. However, the impact of delayed surgery on these outcomes is still controversial, at best, owing to the inconsistency in study populations, methods, and endpoints. Some studies suggest that the delaying RP may even be safe for up to 12 months, while most of the studies reported shorter safe delay periods (i.e., 3–6 months). Indeed, different definitions of delay in the literature led to inconsistency in the results. Consequently, any decision to delay the definitive treatment of patients with intermediate- and/or high-risk PCa will tend to bias in terms of the safe delay time. Moreover, it is important to note that the retrospective nature of most of the selected studies may have led to a selection bias with regards to comorbidities of patients who underwent immediate RP and those who underwent deferred RP. The majority of the studies did not report the reason for the delay. While the delay of a definitive treatment in patients with intermediate- and/or high-risk PCa should be done with caution and based on a rational decision, a 3-month delay may be safe and acceptable. Due to selection bias, elderly patients with more comorbidities could be most likely to be selected for the delayed management than younger patients without comorbidities (possible attrition bias).

The Prostate Cancer EAU Guidelines Panel, which is applicable in the current COVID-19 pandemic, recommends postponing RP until after the pandemic in intermediate- and high-risk PCa patients and does not advocate for treatment with neoadjuvant ADT in most cases [4]. On the other hand, recently, there was an international accelerated consensus on the management of intermediate-risk patients using close surveillance, while high-risk prostate cancer patients may be managed using either immediate surgery in the absence of COVID-19 risk features or alternatively treated using ADT until it is safe to proceed with surgery [29]. According to the present systematic review findings, we could recommend a 3-month delay as a safe delay time in intermediate-risk PCa patients and the same delay with caution for high-risk PCa patients until safe surgical conditions are possible.

Our systematic review is not devoid of limitations. The main limitation is the retrospective and heterogeneous nature of most of the included studies. We found significant heterogeneity across the studies in terms of delay definitions, definitions of outcomes (endpoints), and baseline clinicopathologic features of patients. We were also unable to elaborate on the effect of treatment delay in different PCa risk groups due to the fact that most of the included studies did not report separate analyses based on risk groups. Moreover, performing a quantitative synthesis was not feasible due to different outcomes of interest and heterogeneity in the definition of delayed RP. Therefore, well-designed controlled comparative studies are required to clarify our findings.

## Conclusions

The COVID-19 pandemic is likely to continue affecting the health care system for the foreseeable future. Consequently, it is important to prioritize the timely care of patients with PCa for whom delays are most likely to result in worse oncologic outcomes. According to the present systematic review in patients with intermediate- and high-risk PCa, a delay of RP for up to 3 months is likely to be safe, as it is not associated with biochemical recurrence, worse oncological survival outcomes, other adverse pathologic outcomes, or the need for adjuvant therapy. In addition, because of the long duration of the current pandemic and the fact that it could continue for a longer time period, we recommend further studies to prospectively assess the outcomes secondary to delays in PCa patients care during the current pandemic. This could help elucidate the oncologic impact of delays and prepare for future events that could result in prolonged delays in definitive care in patients with intermediate- and high-risk PCa.
